# Isobutane/2-butene alkylation catalyzed by Brønsted–Lewis acidic ionic liquids†

**DOI:** 10.1039/c8ra03485k

**Published:** 2018-05-29

**Authors:** Shiwei Liu, Shuang Tan, Bing Bian, Hailong Yu, Qiong Wu, Zhiguo Liu, Fengli Yu, Lu Li, Shitao Yu, Xiuyan Song, Zhanqian Song

**Affiliations:** College of Chemical Engineering, Qingdao University of Science and Technology No. 53 Zhengzhou Road Qingdao 266042 China liufusheng63@sina.com liushiweiqust@126.com +86 532 84022719; School of Chemical and Environmental Engineering, Shandong University of Science and Technology Qingdao 266510 China; Key Laboratory of Eco-Chemical Engineering of Ministry of Education, Qingdao University of Science and Technology No. 53 Zhengzhou Road Qingdao 266042 China

## Abstract

The alkylation reaction of isobutane with 2-butene to yield C_8_-alkylates was performed using Brønsted–Lewis acidic ionic liquids (ILs) comprising various metal chlorides (ZnCl_2_, FeCl_2_, FeCl_3_, CuCl_2_, CuCl, and AlCl_3_) on the anion. IL 1-(3-sulfonic acid)-propyl-3-methylimidazolium chlorozincinate [HO_3_S-(CH_2_)_3_-mim]Cl-ZnCl_2 (*x*=0.67)_ exhibited outstanding catalytic performance, which is attributed to the appropriate acidity, the synergistic effect originating from its double acidic sites and the promoting effect of water on the formation and transfer of protons. The Lewis acidic strength of IL played an important role in improving IL catalytic performance. A 100% conversion of 2-butene with 85.8% selectivity for C_8_-alkylate was obtained under mild reaction conditions. The IL reusability was good because its alkyl sulfonic acid group being tethered covalently, its anion [Zn_2_Cl_5_]^−^ inertia to the active hydrogen, and its insolubility in the product. IL [HO_3_S-(CH_2_)_3_-mim]Cl-ZnCl_2_ had potential applicability in the benzene alkylation reaction with olefins and halohydrocarbons.

## Abstract 2

The alkylation reaction of isobutane with 2-butene to yield C_8_-alkylates was performed using Brønsted–Lewis acidic ionic liquids (ILs) comprising various metal chlorides (ZnCl_2_, FeCl_2_, FeCl_3_, CuCl_2_, CuCl, and AlCl_3_) on the anion.

## Graphical abstract

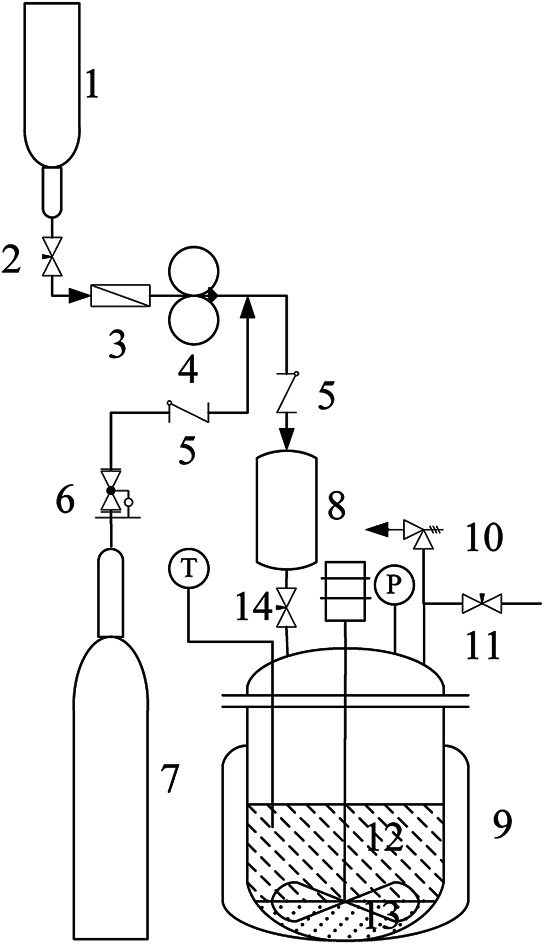

## 1. Introduction

The alkylation reaction of isobutane with C_4_-olefins forms an alkylate consisting of branched paraffin, which is used as a high-octane and environmentally friendly blending component for gasoline.^1,2^ Sulfuric acid and hydrofluoric acid are used currently as catalysts in industry. Both catalysts exhibit some advantages, such as a high reagent conversion and a rather high alkylate quality. They also display some shortcomings, such as a high or extreme toxicity (hydrofluoric acid), corrosivity, harmfulness to the environment, high acid consumption (70–100 kg H_2_SO_4_/ton alkylate), and formation of tars. Current research is focused on the need for less hazardous and more effective catalysts for the reaction. Recently, two main types of solid catalysts have been considered as potential substitutes to overcome problems associated with conventional alkylation catalysts.^3,4^ The first are active material (*e.g.*, BF_3_, La, or 12-tungstophosphoric acid) catalysts adsorbed on inert supports such as alumina or silica^3–5^ and the second are solid acids (*e.g.*, H-USY zeolites) and superacid catalysts based on sulfate-promoted metal oxides (*e.g.*, sulfated zirconia).^6–8^ Both catalysts are active in the alkylation reaction, but they also exhibit some disadvantages, such as a fast deactivation, blockage of small pores, leaching (active material catalysts), incomplete activity recovery, and high active component (active material catalysts) and regeneration (solid acids) costs. Therefore, research focused on alternative catalysts is desired.

As a type of green solvent/catalyst, ionic liquids (ILs) have attracted much attention for applications in chemistry and industry because of their chemical stability, thermal stability, low vapor pressure, and high ionic conductivity properties.^9,10^ ILs, especially acidic ILs, are recognized as promising candidates to overcome shortcomings of industrially employed traditional liquid catalysts and solid catalysts in many reactions, such as esterification, polymerization, and alkylation. Recently, acidic IL catalysts also been scrutinized in the alkylation reaction of isobutane/2-butene.^11–17^ Chloroaluminate-based acidic ILs were often used as catalysts in the alkylation reaction. The conversion of 2-butene reached 91.0%, but the selectivity of C_8_-alkylate was only 50.5% and only 10% trimethylpentane (TMP) selectivity was obtained.^13^ Many other chloroaluminate ILs were also investigated in the alkylation reaction of isobutane/2-butene.^13,14^ However, the amounts of TMP in the alkylation products were seldom greater than 75 wt%. In contrast, the TMP selectivity is close to 80% in commercial alkylation.^1^ To improve the catalytic performance of chloroaluminate IL, HCl or Brønsted acidic IL 1-methyl-3-(4-sulfobutyl)-imidazolium hydrogen sulfate [HO_3_Sbmim][HSO_4_] were used as co-catalysts in the alkylation reaction of isobutane/2-butene.^15,16^ The TMP selectivity increased from 21.3% to 52.7% by addition of 50 wt% Brønsted acidic IL [HO_3_Sbmim][HSO_4_] into IL 1-methyl-3-octyl-imidazolium bromo-chloroaluminate [omim]Br-AlCl_3 (*x*=1.5)_ (*x* represent the molar ratio of AlCl_3_).^16^ A 95.8% C_8_-alkylate selectivity and 90.4% TMP selectivity were achieved when HCl gas was bubbled into the Lewis acidic IL triethylamine hydrochloride–aluminum chloride/cuprous chloride [(C_2_H_5_)_3_NH]Cl–AlCl_3_/CuCl.^17^ These results indicate that the addition of a Brønsted acidic compound was an effective way to improve the catalytic activity and selectivity of Lewis acidic IL. However, chloroaluminate IL hydrolyzes easily to release HCl gas because it is sensitive to moisture, and it is difficult to recover HCl gas together with IL for recycling of the catalytic system. This causes irreversible deactivation of the catalytic system. As a result, the catalytic system can only be applied a few times with good catalytic performance and then its performance decreases in further repetitive use. Therefore, Brønsted–Lewis acidic ILs with a good stability for moisture were synthesized and used in the alkylation reaction of isobutane/2-butene. Compared with chloroaluminate IL and traditional catalysts, the synthesized Brønsted–Lewis acidic ILs were inert to moisture and had a synergistic effect originating from its double acidic sites. This provides IL with an efficient catalytic performance and a good reusability for the alkylation reaction.

## 2. Material and methods

### 2.1. Materials

Isobutane (*i*-butane 95.2 wt%, *n*-butane 3.4 wt%, and others 1.4 wt%) and 2-butene (2-butene 88.5 wt%, butane 3.4 wt%, isobutene 4.5 wt%, 1-butene 2.3 wt%, and others 1.3 wt%) were from Dalian Airichem Specialty Gases & Chemicals Co. Ltd., China. 1-Methylimidazole (99.8 wt%, Zhejiang Kaile Chemicals Co. Ltd., China), 1,3-propane sultone (1,2-oxathiolane-2,2-dioxide, 99.7 wt%, Wuhan Fengfan Chemicals Co. Ltd., China), and other chemicals (analytical purity) were commercially available. All materials were used without further purification.

### 2.2. Preparation of ILs

**Scheme 1:**
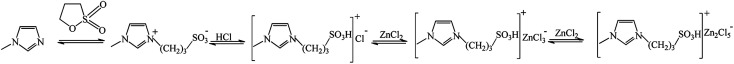
Synthesis of IL [HO_3_S-(CH_2_)_3_-mim]Cl-ZnCl_2 (*x*=0.67)_.

Under vigorous stirring, 0.1 mol 1-methylimidazole was reacted with 0.1 mol 1,3-propane sulfone in 50 mL solvent ethyl acetate for 2 h at 50 °C, and the mixture was filtered to obtain a white precipitate. The precipitate was washed three times with 30 mL ethyl acetate and dried at 100 °C for 2 h, giving 18.6 g 3-(1-methylimidazolium-*N*-yl)-propane-1-sultonate (MIM-PS, yield 91.2%). MIM-PS (0.05 mol) was dissolved in 15 mL water and was reacted with equimolar hydrochloric acid at room temperature for 30 min and then at 90 °C for 2 h, to obtain an aqueous solution of 1-(3-sulfonic acid)-propyl-3-ethyl-imidazolium chloride [HO_3_S-(CH_2_)_3_-mim]Cl. Then 0.1 mol ZnCl_2_ was added to the obtaining aqueous solution and was heated under reflux for 2 h. The water was removed at 1 kPa at 90 °C, to yield 31.4 g IL [HO_3_S-(CH_2_)_3_-mim]Cl-ZnCl_2 (*x*=0.67)_ (Scheme 1). The acidity of the obtained IL depended on the amount of ZnCl_2_ added. When the molar fraction of ZnCl_2_ to [HO_3_S-(CH_2_)_3_-mim]Cl was less than 0.5, IL only possessed Brønsted acidity and no Lewis acidity. Otherwise, IL was Brønsted and Lewis dual acidic. Other acidic ILs [HO_3_S-(CH_2_)_3_-mim]Cl-ZnCl_2 (*x*=0.50, 0.60, 0.71, or 0.75)_, [HO_3_S-(CH_2_)_3_-mim]Cl-CuCl_2 (*x*=0.67)_, [HO_3_S-(CH_2_)_3_-mim]Cl-CuCl _(*x*=0.67)_, [HO_3_S-(CH_2_)_3_-mim]Cl-AlCl_3 (*x*=0.67)_, [HO_3_S-(CH_2_)_3_-mim]Cl-FeCl_3 (*x*=0.67)_, and [HO_3_S-(CH_2_)_3_-mim]Cl-FeCl_2 (*x*=0.67)_ were synthesized according to the same process. IL 1-*n*-butyl-3-methyl-imidazolium chlorozincinate [C_4_mim]Cl-ZnCl_2 (*x*=0.67)_ was synthesized according to the ref. 16.

The water content of the synthesized ILs was tested with a coulometric Karl Fishcher titrator, using a Metrohm 831 KF coulometer, for 0.1 g ILs samples, to ±10 ppm accuracy in water mass content. The ILs dynamic viscosity (*η*) was measured at 0.1 MPa as a function of temperature using an electromagnetic VINCI Tech. EV1000 viscometer. The temperature of the measurement chamber was controlled by an external circulating bath and measured inside the chamber by a platinum resistance probe to ±0.01 °C. Measurements were carried in the 25 to 98 °C temperature range. The calibration of the viscometer was done using certified oils by Koehler Inc. The ILs density was measured using pycnometer. The calibration of pycnometer was done with millipore water. Temperature was kept constant within ±0.1 °C using PID controller and circulating water using thermo static-fluid bath. The partition coefficient of IL in the *n*-octanol/water system (log Kow) was determined at 37 °C by the shake-flask method. The structure of ILs was confirmed using a Nicolet 510P FT-IR spectrometer and a Bruker AV500 NMR spectrometer. The acidities of ILs were characterized and determined on the basis of the Hammett acidity function. As shown in the following equation, the Hammett acidity function is expressed as: *H*_0 =_ p*K*(I)_aq_ + log([I]/[IH]^+^). Where p*K*(I)_aq_ is the p*K*a value of the indicator *p*-nitroaniline referred to as the aqueous solution, [IH]^+^ and [I] are respectively the molar concentrations of the protonated and unprotonated forms of the indicator, which can usually be determined by UV-visible spectroscopy.^18^

MIM-PS:IR (KBr disc): *ν* 3464, 3199, 2989, 1629, 1485, 1454, 1222, 1149, 1041, 735, 603, 518. ^1^H NMR (500 MHz, D_2_O): *δ* 8.70 (s, 1H), 7.49 (s, 1H), 7.40 (s, 1H), 4.30 (t, 2H), 3.81 (s, 3H), 2.85 (t, 2H), 2.25 (m, 2H). ^13^C NMR (125 MHz, D_2_O): *δ* 135.82, 123.50, 121.85, 47.41, 46.97, 35.46, 24.73. [HO_3_S-(CH_2_)_3_-mim]Cl: water content: 257 ppm. Density (25 °C): 1.024 g cm^−3^. log Kow: −1.87. IR (KBr disc): *ν* 3382, 3142, 3117, 1720, 1653, 1572, 1227, 1171, 1029, 807, 592. ^1^H NMR (500 MHz, D_2_O): *δ* 8.53 (s, 1H), 7.32 (s, 1H), 7.25 (s, 1H), 4.16 (t, 2H), 3.71 (s, 3H), 2.71 (t, 2H), 2.11 (m, 2H). ^13^C NMR (500 MHz, D_2_O): *δ* 134.72, 123.31, 122.16, 47.75, 46.92, 35.26, 24.73. [HO_3_S-(CH_2_)_3_-mim]Cl-ZnCl_2 (*x*=0.67)_: water content: 246 ppm. Density (25 °C): 2.367 g cm^−3^. log Kow: −2.32. IR (KBr disc): *ν* 3454, 3156, 3121, 2969, 1625, 1583, 1469, 1252, 1168, 1051, 837, 756, 625, 531. ^1^H NMR (500 MHz, DMSO): *δ* 8.51 (s, 1H), 7.27 (s, 1H), 7.21 (s, 1H), 4.10 (t, 2H), 3.63 (s, 3H), 2.65 (t, 2H), 2.06 (m, 2H). ^13^C NMR (500 MHz, DMSO): *δ* 135.81, 121.28, 120.04, 46.57, 45.86, 34.32, 23.35. [HO_3_S-(CH_2_)_3_-mim]Cl-FeCl_2 (*x*=0.67)_: water content: 185 ppm. Density (25 °C): 2.738 g cm^−3^. log Kow: −1.94. IR (KBr disc): *ν* 3382, 3125, 3114, 2989, 1629, 1579, 1485, 1148, 1041, 837, 735, 603, 528. ^1^H NMR (500 MHz, DMSO): *δ* 8.55 (s, 1H), 7.42 (s, 1H), 7.35 (s, 1H), 4.26 (t, 2H), 3.81 (s, 3H), 2.74 (t, 2H), 2.21 (m, 2H). ^13^C NMR (500 MHz, DMSO): *δ* 134.72, 122.31, 121.16, 47.25, 46.92, 34.26, 24.73. [HO_3_S-(CH_2_)_3_-mim]Cl-FeCl_3 (*x*=0.67)_: water content: 220 ppm. Density (25 °C): 2.407 g cm^−3^. log Kow: −2.30. IR (KBr disc): *ν* 3477, 3159, 3116, 2987, 1623, 1575, 1469, 1227, 1170, 1048, 835, 747, 621, 525. ^1^H NMR (500 MHz, DMSO): *δ* 8.47 (s, 1H), 7.19 (s, 1H), 7.23 (s, 1H), 3.97 (t, 2H), 3.54 (s, 3H), 2.55 (t, 2H), 1.92 (m, 2H). ^13^C NMR (500 MHz, DMSO): *δ* 136.71, 124.30, 123.13, 48.727, 47.896, 36.22, 25.71. [HO_3_S-(CH_2_)_3_-mim]Cl-CuCl_2 (*x*=0.67)_: Water content: 147 ppm. Density (25 °C): 2.557 g cm^−3^. log Kow: −2.35. IR (KBr disc): *ν* 3434, 3157, 2976, 1715, 1575, 1463, 1232, 1173, 1034, 878, 749, 622, 579. ^1^H NMR (500 MHz, DMSO): *δ* 8.47 (s, 1H), 8.35 (s, 1H), 7.25 (s, 1H), 4.16 (t, 2H), 3.71 (s, 3H), 2.71 (t, 2H), 2.11 (m, 2H). ^13^C NMR (500 MHz, DMSO): *δ* 134.75, 123.34, 122.12, 47.73, 46.94, 35.28, 24.75. [HO_3_S-(CH_2_)_3_-mim]Cl-CuCl _(*x*=0.67)_: water content: 181 ppm. Density (25 °C): 2.832 g cm^−3^. log Kow: −2.33. IR (KBr disc): *ν* 3458, 3140, 2992, 1720, 1568, 1456, 1223, 1169, 1017, 882, 755, 638, 581. ^1^H NMR (500 MHz, DMSO): *δ* 8.56 (s, 1H), 8.43 (s, 1H), 7.37 (s, 1H), 4.30 (t, 2H), 3.81 (s, 3H), 2.86 (t, 2H), 2.34 (m, 2H). ^13^C NMR (500 MHz, DMSO): *δ* 138.56, 124.32, 120.12, 46.80, 44.87, 34.36, 22.42.

### 2.3. Alkylation apparatus and process

**Fig. 1:**
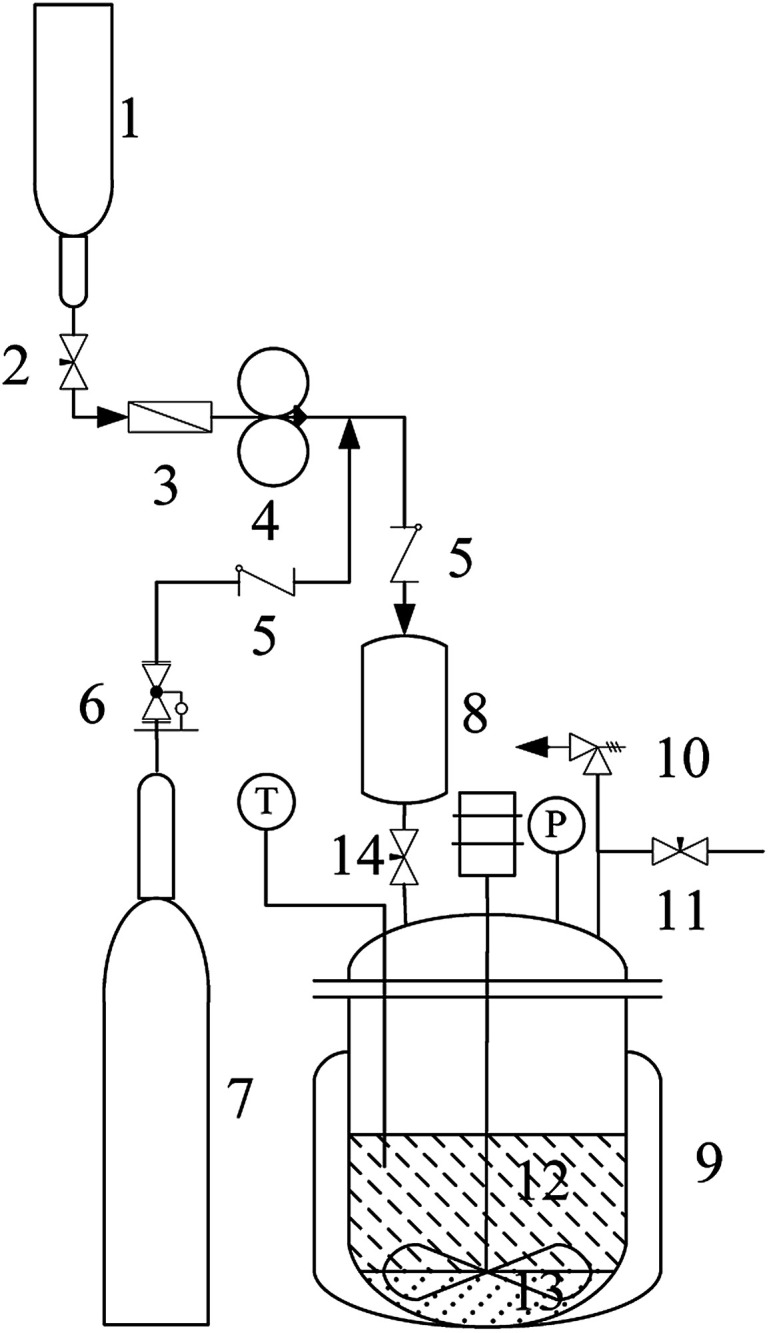
Apparatus for the isobutane/2-butene alkylation reaction. (1) Feed storage tank, (2) needle valve, (3) filter, (4) double-piston metering pumps, (5) one-way valve, (6) pressure relief valve group, (7) constant pressure nitrogen, (8) feed tank, (9) reactor, (10) anti-exploding valve, (11) air-vent, (12) reaction mixture, (13) catalytic phase, (14) needle valve.

The alkylation apparatus is shown in Fig. 1. Reactions were carried out in batch mode in a 75 mL pressure reactor with stirring at 600 rpm. Before the experiment, a mixture (11.3 g) of isobutane and 2-butene at a certain molar ratio (I/O = 10 : 1) and stored in the feed storage tank (1) was pumped into the feed tank (8) using a double-piston metering pumps (4). The catalyst-containing IL [HO_3_S-(CH_2_)_3_-mim]Cl-ZnCl_2 (*x*=0.67)_ (3.0 g) and water (1.3 g) were added into the reactor (9). The reactor was sealed and air was excluded using the high-pressure nitrogen. After stirring, the reactor was heated to 80 °C, and the high-pressure nitrogen (6) and feed tank (14) valves were opened. The mixture of isobutane and 2-butene in the feed tank (8) was forced into the reactor and reacted for 4 h at 80 °C. The pressure in the autoclave was maintained at 2.0 MPa using high-pressure nitrogen to keep the reactant and the product in the liquid phase during the reaction. After the reaction was complete, the reactor was cooled in an ice water bath. The reactor pressure was relieved through an airvent (11) and all excluded gas was collected into a gasbag to determine the mass (0.2 to0.1 g) of 2-butene and isobutane by gas chromatography (GC) using CH_3_Cl as the internal standard. The conversion of 2-butene was calculated by: *C*% = *m*_1_(2-butene)/*m*(2-butene) ut100, where *m*_1_(2-butene) and *m*(2-butene) were the mass of 2-butene consumed in the reaction and feed of 2-butene, respectively. The liquid phase separated into two phases because of the product insolubility in the catalytic system. The upper layer (10.9 in0.1 g) was decanted from the catalyst layer and was analyzed by GC to determine the result of the alkylation reaction. The product selectivity was calculated from *W*_S_/*W*_ALL_ × 100, where *W*_S_ is the amount of one of the products and *W*_ALL_ is the total amount of products, including hydrocarbons with five to seven carbon atoms (C_5–7_), TMP, dimethylhexane (DMH), hydrocarbons with more than nine carbon atoms (C_9_^+^), and others. The IL layer (4.3 , 0.1 g) was reused directly in the recycle experiments to establish its reusability. When solid catalysts such as AlCl_3_ and ZnCl_2_ were used in the alkylation, they were directly added into the pressure reactor in glove box, and other operations were similar to the above process. All experiments were repeated five times to determine the reproducibility of the results.

The gas and upper layer of the liquid samples were characterized qualitatively using GC-MS on a HP6890/5973 GC-MS equipped with a PONA capillary column (50 m × 0.25 mm × 0.25 μm) and their quantitative analyses were determined using GC on a HP6890 GC equipped with a PONA capillary column (50 m × 0.25 mm × 0.25 μm). The injector and detector temperatures were 250 °C and 300 °C, respectively. The temperature program of the GC oven was as follows: hold at 40 °C for 2 min, increase to 60 °C at 2°C min^−1^, increase to 120 °C at 1°C min^−1^, increase to 180 °C at 2°C min^−1^, and finally, hold at 180 °C for 15 min. Qualitative analysis was conducted based on the holding time of the peak and the contents of the reactants and products were indicated by the GC ChemStation system according to the area of each chromatograph peak.

## 3. Results and discussion

### 3.1. Effect of different catalysts on reaction results

**Fig. 2:**
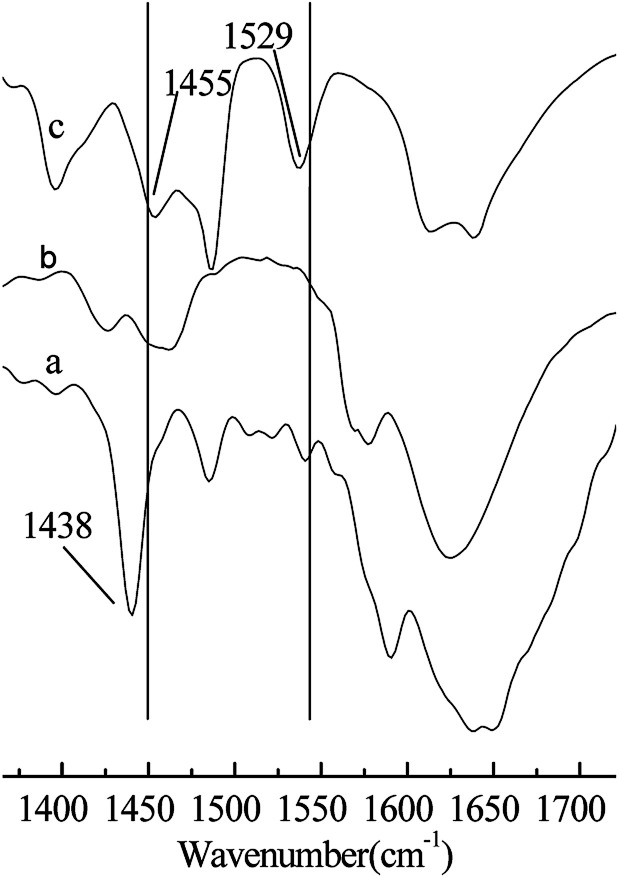
FT-IR spectra of the samples using pyridine as a probe. (a) Pure pyridine. (b) [HO_3_S-(CH_2_)_3_-mim]Cl-ZnCl_2 (*x*=0.67)_. (c) Pyridine/[HO_3_S-(CH_2_)_3_-mim]Cl-ZnCl_2 (*x*=0.67)_. *V*(pyridine) : *V*(IL) = 1 : 2.

As shown in Table 1, when H_2_SO_4_ was used as a catalyst in the alkylation reaction (Entry 1), the conversion of 2-butene was 100%, but most of the product was C_5–7_ and C_9_^+^ hydrocarbons generated from the cracking and oligomerization reaction, respectively. This may be because the catalytic activity of H_2_SO_4_ is too strong. It has been reported that there is an induction period before the alkylation reactions starts, the duration of the period is inversely related to the acid strength and may affect the formation of reaction precursors.^19^ The C_8_-alkylate product can be cracked or react further with 2-butene because of the back-mixing problem. When AlCl_3_ was used as a catalyst in the alkylation reaction, the conversion of 2-butene was 100%, and the selectivity of TMP was 49.6% (Entry 2). AlCl_3_ exhibits no good catalytic performance for the alkylation reaction of isobutane/2-butene, although its catalytic performance is excellent for many other alkylation reactions. When Brønsted acidic IL [HO_3_S-(CH_2_)_3_-mim]Cl or Lewis acid ZnCl_2_ were used as catalysts (Entries 3 and 4), the alkylation reaction results were poor. This is attributed to a low acid strength for catalysts that cannot trigger alkylation reaction to begin, which results in their poor catalytic performances. When Brønsted–Lewis acidic IL [HO_3_S-(CH_2_)_3_-mim]Cl-ZnCl_2_ was used, the conversion of 2-butene was 100%, the selectivity of TMP reached 80.5%, and TMP/DMH reached 15.2 (Entry 6), which indicates that Brønsted–Lewis acidic IL [HO_3_S-(CH_2_)_3_-mim]Cl-ZnCl_2_ was an efficient catalyst in the alkylation reaction. The good catalytic performance may be attributed to the appropriate acid strength of the IL and the synergistic effect originating from the Brønsted and Lewis acids of IL. Both are responsible for the ability of IL to protonate the olefin.^20^ As shown in Table 1, Hammett acidity of the [HO_3_S-(CH_2_)_3_-mim]Cl-ZnCl_2_ and [HO_3_S-(CH_2_)_3_-mim]Cl-FeCl_3_ were less than the others (Entries 6–11), indicating that the acid strength of both is stronger. So their abilities to protonate olefin are stronger and the conversion of 2-butene was higher. Otherwise, to confirm the synergistic effect on the catalytic performance, Brønsted acidic IL [HO_3_S-(CH_2_)_3_-mim]Cl and Lewis acidic IL [C_4_mim]Cl-ZnCl_2_ were selected and used as catalysts for comparison (Entries 3 and 5). The results show that both catalytic performances were not as good as that of IL [HO_3_S-(CH_2_)_3_-mim]Cl-ZnCl_2_ (Entry 6), which suggests that the synergistic effect originates from the Brønsted and Lewis acids of IL and is important in the alkylation reaction. To clarify the Brønsted and Lewis acids of IL [HO_3_S-(CH_2_)_3_-mim]Cl-ZnCl_2_, the acidity of IL was determined using Fourier transform infrared (FT-IR) spectroscopy with pyridine as the probe,^21^ and the results are shown in Fig. 2. Compared with the FT-IR spectra of pure pyridine (Fig. 2a) and IL [HO_3_S-(CH_2_)_3_-mim]Cl-ZnCl_2 (*x*=0.67)_ (Fig. 2b), the FT-IR spectrum of pyridine/[HO_3_S-(CH_2_)_3_-mim]Cl-ZnCl_2 (*x*=0.64)_ had two new characteristic absorption peaks at 1529 cm^−1^ and 1455 cm^−1^ (Fig. 2c). Both were characteristic absorption peaks of the [PyH]^+^ cation and the Py–Lewis complex, which form from the reactions of pyridine with the Brønsted and Lewis acidic centers, respectively.^22^ This result indicates that [HO_3_S-(CH_2_)_3_-mim]Cl-ZnCl_2 (*x*=0.67)_ was Brønsted and Lewis acidic.^23^

**Table 1:** Effect of the different catalysts on the alkylation reaction results^a^

Entry	Catalyst	*H*_0_	*C*/%	Selectivity/%				TMP/DMH
				C_5–7_	TMP	DMH	C_9_^+^	
1	H_2_SO_4_^b^	−11.95	100	18.5	22.6	8.5	44.2	2.7
2	AlCl_3_^b^	−2.60	100	18.7	49.6	12.3	15.2	4.0
3	[HO_3_S-(CH_2_)_3_-mim]Cl	5.65	51.3	4.3	64.7	15.5	8.9	4.2
4	ZnCl_2_^c^	—	10.3	—	—	—	—	
5	[C_4_mim]Cl-ZnCl_2_	—	66.0	9.6	64.1	7.8	10.6	8.2
6	[HO_3_S-(CH_2_)_3_-mim]Cl-ZnCl_2_	1.26	100	5.2	80.5	5.3	6.2	15.2
7	[HO_3_S-(CH_2_)_3_-mim]Cl-CuCl_2_	2.52	54.2	4.1	62.3	10.3	18.6	6.0
8	[HO_3_S-(CH_2_)_3_-mim]Cl-CuCl	2.86	48.9	4.3	58.0	10.2	21.4	5.7
9	[HO_3_S-(CH_2_)_3_-mim]Cl-AlCl_3_	−2.42	25.9	—	—	—	—	
10	[HO_3_S-(CH_2_)_3_-mim]Cl-FeCl_3_	0.82	100	9.6	71.5	8.2	7.9	8.7
11	[HO_3_S-(CH_2_)_3_-mim]Cl-FeCl_2_	1.65	70.3	6.2	68.9	8.6	10.2	8.0
12^c^	[HO_3_S-(CH_2_)_3_-mim]Cl-ZnCl_2_	—	66.8	5.9	72.3	6.2	12.1	11.7
13^c^	[HO_3_S-(CH_2_)_3_-mim]Cl-FeCl_3_	—	58.0	12.6	66.5	7.6	10.8	8.8
14^c^	[HO_3_S-(CH_2_)_3_-mim]Cl-AlCl_3_	—	65.2	17.2	39.6	9.0	29.1	4.4

a Feed 11.3 g, I/O = 10 : 1, catalyst 3.0 g, *x* (Lewis acidic metal chloride) = 0.67, H_2_O 1.3 g, *T* = 80 °C, *t* = 4 h.

b *T* = 0 °C, H_2_O 0 g, the other conditions were the same as footnote *a*.

c H_2_O 0 g, the other conditions were the same as footnote *a*. *H*_0_ represent Hammett acidity.

Among the results catalyzed by the Brønsted–Lewis acidic ILs (Entries 6–11), the metal chlorides had a decisive influence on the catalytic performance of ILs. IL [HO_3_S-(CH_2_)_3_-mim]Cl-ZnCl_2_ showed the best catalytic performance (Entry 6), and the others were poor. This is because of the difference in IL acidic strength which is responsible for the IL ability to protonate the 2-butene. With increasing Lewis acidity of the metal chloride, IL Lewis acidity becomes stronger, and results in a higher IL catalytic activity.^24^ When IL [HO_3_S-(CH_2_)_3_-mim]Cl-CuCl_2_ and [HO_3_S-(CH_2_)_3_-mim]Cl-CuCl were used in the alkylation, the conversion of 2-butene was less than 55%, and the selectivity of C_9_^+^ was more than 18% (Entries 8 and 9). This is because the catalytic activity of IL was too low, so the oligomerization of 2-butene occurred easily. When IL [HO_3_S-(CH_2_)_3_-mim]Cl-FeCl_3_ was used, the conversion of 2-butene was 100%, but the selectivity of TMP was only 71.5%, and the selectivities of C_5–7_ and C_9_^+^ were 9.6% and 7.9%, respectively (Entry 10). This may occur because the Lewis acid strength of IL [HO_3_S-(CH_2_)_3_-mim]Cl-FeCl_3_ is too strong, and improves the cracking and oligomerization reaction. Using IL [HO_3_S-(CH_2_)_3_-mim]Cl-FeCl_2_ as catalyst, the conversion of 2-butene was only 70.3%, and the selectivity of TMP was 68.9%, indicating its poor catalytic performance (Entry 11). This maybe because of its low acid strength to make it hard to trigger alkylation reactions begins. As a result, the alkylation reaction is difficult to proceed smoothly, and the oligomerization reaction increased. The above results indicate that too strong or too low an acid strength is unfavorable for TMP formation. The optimum acid value is between 0.82–1.65.

**Scheme 2:**
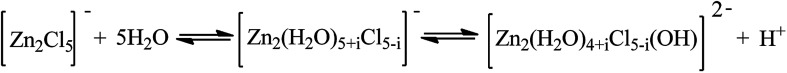
The formation of the dioctahedral complex from the reaction of anion [Zn_2_Cl_5_]^−^ and water.

Interestingly, the addition of water to IL [HO_3_S-(CH_2_)_3_-mim]Cl-ZnCl_2_ catalyst promoted the alkylation reaction, Although water reduces the acid strength of ILs by means of dilution and formation of a complex with metal ions (see ESI materials Table S2†). When no water was used, the conversion of 2-butene was less than 70% (Entry 12), but when 30 wt% water was added to IL, the 2-butene conversion reached 100% (Entry 6). A similar result was obtained when IL [HO_3_S-(CH_2_)_3_-mim]Cl-FeCl_3_ was used (Entries 10 and 13). This maybe because the addition of water to IL reducing the viscosity of IL and increasing the isobutane and olefin solubility in IL (see ESI material Fig. S1 and Table S1†), which increases the rate of mass and the proton transfer. So the formation of carbenium ions is enchanced. Otherwise, the anion [Zn_2_Cl_5_]^−^ of IL maybe react with the water to form the dioctahedral complex,^25^ which increases the proton concentration in IL phase (Scheme 2). As a result, the formation of the carbocation and the transfer of proton between carbocation and isobutane maybe accelerate. However, the result was poor in the presence of IL [HO_3_S-(CH_2_)_3_-mim]Cl-AlCl_3_ when the same amount of water was added (Entry 9). This occurs because the AlCl_3_ can react with water and the Brønsted acidic center and release HCl, so the Lewis acidic center and even the Brønsted acidic center of IL were lost. This also explains why the result was unsatisfactory for the catalyst [HO_3_S-(CH_2_)_3_-mim]Cl-AlCl_3_ when water was not added (Entry 14).

**Fig. 3:**
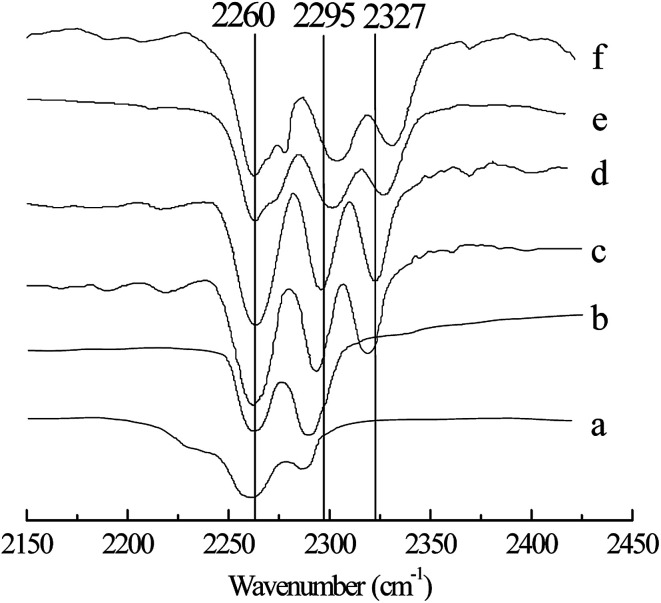
FT-IR spectra of the ILs using acetonitrile as a probe. (a) Pure acetonitrile, (b) acetonitrile/[HO_3_S-(CH_2_)_3_-mim]Cl-ZnCl_2 (*x*=0.50)_. (c) Acetonitrile/[HO_3_S-(CH_2_)_3_-mim]Cl-ZnCl_2 (*x*=0.60)_. (d) Acetonitrile/[HO_3_S-(CH_2_)_3_-mim]Cl-ZnCl_2 (*x*=0.67)_. (e) Acetonitrile/[HO_3_S-(CH_2_)_3_-mim]Cl-ZnCl_2 (*x*=0.71)_. (f) Acetonitrile/[HO_3_S-(CH_2_)_3_-mim]Cl-ZnCl_2 (*x*=0.75)_. *V*(acetonitrile) : *V*(IL) = 1 : 2 in the samples of (b–f).

Table 2 shows the effect of *x*(ZnCl_2_) on the alkylation reaction. The conversion of 2-butene and the selectivity of TMP increased with increasing *x*(ZnCl_2_) (Entries 1–5). When *x*(ZnCl_2_) was 0.67, the alkylation of isobutane/2-butene was catalyzed. This may be explained by the fact that, when *x*(ZnCl_2_) is greater than 0.50, IL is a Brønsted and Lewis acidic, and by increasing *x*(ZnCl_2_), the Lewis acidity of IL increases,^22^ which enhances IL catalytic activity. However, a further increase in *x*(ZnCl_2_) was unfavorable for the alkylation reaction. When *x*(ZnCl_2_) reached 0.71, the TMP selectivity decreased to 72.4%, and the selectivities of C_5–7_ and C_9_^+^ increased to 7.9% and 11.0%, respectively (Entry 4). When *x*(ZnCl_2_) was 0.75, the TMP selectivity decreased to 58.5%, and the selectivities of C_5–7_ and C_9_^+^ increased to 12.3% and 18.6%, respectively (Entry 5). The above results may be because IL catalytic activity is too high when *x*(ZnCl_2_) reaches more than 0.71, which improves the cracking and oligomerization reaction. To clarify the effect of *x*(ZnCl_2_) on IL [HO_3_S-(CH_2_)_3_-mim]Cl-ZnCl_2_, the Lewis acidity of IL was determined using FT-IR spectroscopy with acetonitrile as the probe,^26^ and the results are shown in Fig. 3. When *x*(ZnCl_2_) was 0.60, a new absorption peak appeared at 2318 cm^−1^ in the spectrum (Fig. 3c), which is the characteristic absorption peak of the CN–Lewis complex that originated from a reaction of the C≡N group of acetonitrile and the Lewis acidic center. With increasing *x*(ZnCl_2_) to 0.71, the characteristic absorption peak moved to 2327 cm^−1^ (Fig. 3e). When *x*(ZnCl_2_) to 0.75, the characteristic absorption peak was 2331 cm^−1^ (Fig. 3f) which indicates that the IL Lewis acidity was strengthened.

**Table 2:** Effect of *x*(ZnCl_2_) on the alkylation reaction results^a^

Entry	*x*(ZnCl_2_)	*H*_0_	*C*/%	Selectivity/%				TMP/DMH
				C_5–7_	TMP	DMH	C_9_^+^	
1	0.50	2.87	61.2	5.3	63.2	14.3	14.2	4.4
2	0.60	1.73	86.4	4.6	75.4	8.5	8.9	8.9
3	0.67	1.26	100	5.2	80.5	5.3	6.2	15.2
4	0.71	0.20	100	7.9	72.4	6.4	11.0	11.3
5	0.75	−1.64	100	12.3	58.5	8.0	18.6	7.3

a Feed 11.3 g, I/O = 10 : 1, catalyst [HO_3_S-(CH_2_)_3_-mim]Cl-ZnCl_2_ 3.0 g, H_2_O 1.3 g, *T* = 80 °C, *t* = 4 h.

**Fig. 4:**
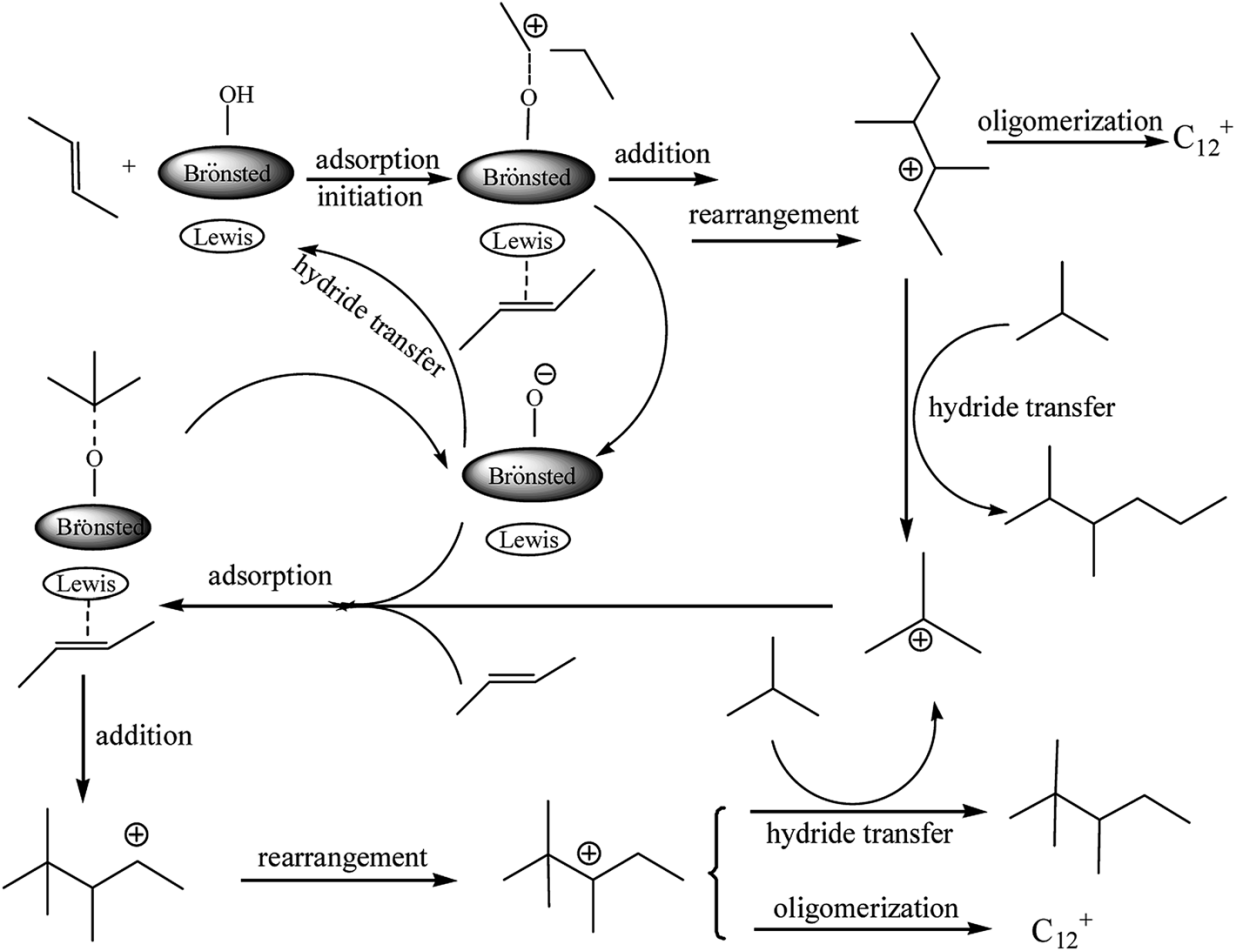
Proposed mechanism of the alkylation with Brønsted–Lewis acidic IL.

Based on the reaction results, a mechanism for the alkylation of isobutane/2-butene in the presence of the Brønsted–Lewis acidic IL was proposed (see Fig. 4) and the synergistic effect between double acidic sites was investigated. 2-Butene was protonated and adsorbed on the IL Brønsted and Lewis acid sites, respectively. On the Brønsted acidic site, a carbocation from 2-butane formated which was necessary to initiate the alkylation reaction. On the Lewis site, the high electronegativity of the π-electron cloud of 2-butene coordinated weakly with the Lewis acid site, so the adsorption of both occurred. The carbocation addition reaction and the adsorbed 2-butene occurred to yield the *sec*-carbocation. A tertiary carbocation with good stability was formed by a rearrangement reaction. Thereafter, the transfer reaction of the negative hydrogen between the tertiary carbocation and isobutane occurred, and DMH and tertiary carbocation of isobutene formed. The tertiary carbocation initiated the next round of alkylation reactions and yielded TMP. The product of C_12_^+^ was also formed by the oligomerization reaction of the C_8_ carbocation with 2-butene. The proposed mechanism shows that Lewis and Brønsted acidities play a synergistic role in the alkylation of isobutane/2-butene.

### 3.2. Effect of reaction conditions on reaction results

Table 3 shows the effect of reaction conditions on the alkylation reaction. The conversion of 2-butane and TMP/DMH increased with increasing IL [HO_3_S-(CH_2_)_3_-mim]Cl-ZnCl_2 (*x*=0.67)_ dosage from 1.5 g to 3.0 g (Entries 1 and 2). When IL dosage was 3.0 g, the conversion of 2-butene was 100%. However, when IL dosage was 4.5 g, TMP/DMH was 16.3, but the selectivity of TMP decreased to 73.4%, and the selectivities for C_5–7_ and C_9_^+^ increased to 10.2% and 9.6%, respectively (Entry 3). This is attributed to too much catalyst usage that increases the number of catalytic active centers, the dissolved quantity and mass transfer of reactants in ILs. So the cracking and oligomerization reactions were favored. The mass fraction of water in ILs had an obvious influence on the reaction (Entries 2, 4–6). Upon increasing the water amount in IL, the results of the alkylation reaction were more satisfying. When the mass fraction of water in IL was more than 30 wt%, the results of the alkylation reaction became poor (Entry 6). This may be that the water use decreased the acid strength and the viscosity of IL (see ESI material Fig. S1 and Table S2†), which improves the mass transfer of reactants and the formation of the TMP. Otherwise, the water maybe react with the anion Zn_2_Cl_5_^−^ of IL to form the dioctahedral complex, which promotes the formation of protons (Scheme 2). The reaction temperature had a significant effect on the alkylation reaction (Entries 2, 7 and 8). The alkylation reaction of isobutane/2-butene is exothermic, so a low reaction temperature favors the formation of C_8_-alkylate and TMP and also inhibits the side reactions. When the reaction temperature was 70 °C, the total selectivity of the C_8_-alkylate was more than 90%, but the conversion of 2-butene was only 86.7% (Entry 7). It is indicated the alkylation reaction did not react completely. This maybe because that, when the reaction temperature was low, the mass transfer of the reagents and the reaction speed are low. The conversion of 2-butene increased with increasing reaction temperature. When the reaction temperature reached 80 °C (Entry 2), 2-butene had reacted completely, and TMP/DMH was 15.2. The results were satisfactory. After that, the selectivity of TMP decreased to 64.1%, TMP/DMH was 14.9, and the selectivities of C_5–7_ and C_9_^+^ were more than 11% (Entry 8). This indicates that a high reaction temperature accelerates side reactions such as the cracking and oligomerization reaction, and results in an increase in selectivities for C_5–7_ and C_9_^+^. Therefore, the optimal reaction temperature was 80 °C. The effect of reaction time on the reaction is also shown in Table 3 (Entries 2, 9 and 10). When the reaction time was 3 h, the conversion of 2-butene was 86.7% and the reaction was not complete. The alkylation reaction was complete at 4.0 h (Entry 2). With prolonged reaction time, the selectivity for TMP decreased to 73.0%, and the C_5–7_ and C_9_^+^ selectivities increased to 7.2% and 12.4%, respectively (Entry 10). This may be because of the TMP cracks and oligomerization for a longer reaction time, which forms C_5–7_ and C_9_^+^ products. So the optimal reaction time was 4.0 h. Some literature reports that the alkylation reaction of isobutane/2-butene occured easily from −5 °C to 15 °C within a short time.^1^ This is attributed to the difference in acidic strength of the used catalyst. Catalysts used in the literature were chloroaluminate ILs with a super acidic catalyst, and its catalytic activity was higher than that of IL used in this paper. However, chloroaluminate ILs are usually sensitive to moisture and hydrolyze easily to form HCl, which results in the irreversible deactivation of the catalyst system.^27^ The effect of I/O molar ratio on the reaction results is also given in Table 3 (Entries 2, 11 and 12). With increasing I/O molar ratio, the 2-butene conversion and TMP selectivity increased, and the C_9_^+^ selectivity decreased. This indicates that increasing I/O ratio favors TMP formation, but disfavors the formation of light and heavy ends.^28^ Based on the above results, the optimal reaction conditions were obtained as follows: feed 11.3 g, I/O = 10 : 1, IL [HO_3_S-(CH_2_)_3_-mim]Cl-ZnCl_2 (*x*=0.67)_ 3.0 g, H_2_O 1.3 g, reaction temperature 80 °C, and reaction time 4 h. Under the above reaction conditions, the conversion of 2-butene and the TMP selectivity were 100% and 80.5%, respectively. And TMP/DMH was more than 15.2.

**Table 3:** Effect of the reaction conditions on the alkylation reaction results^a^

Entry	IL/g	Water/wt%	*T*/°C	*t*/*h*	I/O	*C*/%	Selectivity/%				TMP/DMH
							C_5–7_	TMP	DMH	C_9_^+^	
1	1.5	30	80	4.0	10	88.5	3.2	83.2	6.9	2.8	12.1
2	3.0	30	80	4.0	10	100	5.2	80.5	5.3	6.2	15.2
3	4.5	30	80	4.0	10	100	10.2	73.4	4.5	9.6	16.3
4	3.0	10	80	4.0	10	100	8.3	72.4	4.9	11.4	14.8
5	3.0	20	80	4.0	10	100	6.4	77.9	5.4	7.5	14.4
6	3.0	40	80	4.0	10	91.2	3.6	81.3	7.7	4.6	10.6
7	3.0	30	70	4.0	10	86.7	2.0	83.8	6.4	5.3	13.1
8	3.0	30	90	4.0	10	100	11.3	64.1	4.3	17.6	14.9
9	3.0	30	80	3.0	10	86.7	4.1	82.4	5.5	5.5	15.0
10	3.0	30	80	5.0	10	100	7.2	73.0	4.5	12.4	16.2
11	3.0	30	80	4.0	5	100	6.4	62.5	18.2	10.2	3.4
12	3.0	30	80	4.0	15	100	6.0	82.1	5.1	4.2	16.1

a Feed 11.3 g, catalyst [HO_3_S-(CH_2_)_3_-mim]Cl-ZnCl_2 (*x*=0.67)_, H_2_O 1.3 g.

### 3.3. Reusability of IL

**Fig. 5:**
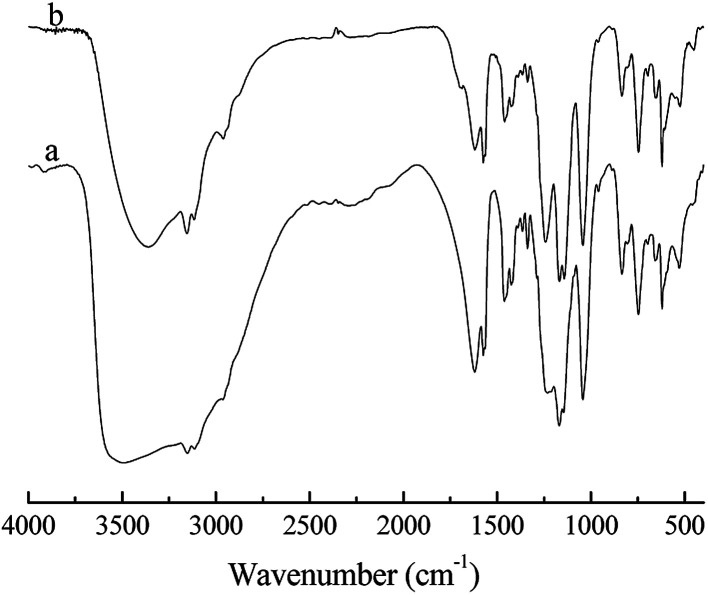
FT-IR spectra of [HO_3_S-(CH_2_)_3_-mim]Cl-ZnCl_2 (*x*=0.67)_. (a) The unused IL. (b) The seven repeatedly used IL.

To investigate the possibility of recycling the IL phase, recycling experiments were conducted under optimized reaction conditions and the results are shown in Table 4. The IL [HO_3_S-(CH_2_)_3_-mim]Cl-ZnCl_2 (*x*=0.67)_ was reused seven times without any obvious decrease in reaction conversion and product selectivity. Therefore, the IL phase showed an excellent reusable performance in the alkylation reaction. The good reusability of the IL phase is attributed to the alkyl sulfonic acid group being tethered covalently to IL cation and the Lewis acidic center of IL being inert and stable to water or the alkyl sulfonic acid group. Fig. 5 shows the IR spectra of the unused IL [HO_3_S-(CH_2_)_3_-mim]Cl-ZnCl_2 (*x*=0.67)_ and the seven repeatedly used IL [HO_3_S-(CH_2_)_3_-mim]Cl-ZnCl_2 (*x*=0.67)_. The peaks in both spectra are similar, which indicates that IL structure was unchanged even after seven cycles. The IR result confirms its good stability. Additionally, the product and IL phase are mutually insoluble and easily separated. The catalyst is not lost easily in the separation process, which is supported by the water/*n*-octanol partition coefficient of IL (the partition coefficient of the IL in the water/*n*-octanol system at 37 °C was 207.3). Meanwhile, it is confirmed that the heavy products were almost no retained in catalytic phase of IL after its several runs. Therefore, IL and its acid active sites are not easily lost and its reusability is good.

**Table 4:** Reusability of IL [HO_3_S-(CH_2_)_3_-mim]Cl-ZnCl_2 (*x*=0.67)_

Entry	*C*/%	Selectivity/%				TMP/DMH
		C_5–7_	TMP	DMH	C_9_^+^	
1	100	5.2	80.5	5.3	6.2	15.2
2	100	5.5	82.4	5.4	4.4	15.3
3	100	5.3	81.5	5.1	5.5	16.0
4	100	5.7	78.2	6.0	7.2	13.0
5	100	4.8	80.8	6.3	5.6	12.8
6	100	6.0	77.6	7.5	5.7	10.3
7	100	4.5	80.3	6.2	6.6	13.0

### 3.4. General applicability of IL catalytic performance for benzene alkylation reactions

To illustrate the general applicability of IL [HO_3_S-(CH_2_)_3_-mim]Cl-ZnCl_2 (*x*=0.67)_ in other alkylation reactions, it was used to catalyze the alkylation reaction of benzene. Table 5 shows the alkylation reactions results. The kinds of alkylation reagents had an obvious influence on the reaction results. When olefins and halohydrocarbons were used as alkylation reagents, the conversion of benzene was nearly 100%, and the target product selectivities were also satisfying (Entries 2–8). In the alkylation reactions of propylene, benzyl chloride, and 2-chloropropane, the selectivities for the target products were greater than 90%. To clarify the synergistic effect of the Brønsted and Lewis acids on IL catalytic performance, the alkylation reaction of benzene with propylene was selected as the model reaction (Entries 10 and 11). When ILs [HO_3_S-(CH_2_)_3_-mim]Cl and [C_4_mim]Cl-ZnCl_2_ were used as catalysts, the benzene conversion was less than 80%, which indicates that both ILs exhibit poor catalytic performance in alkylation. So the synergistic effect can improve the catalytic performance of IL. When methanol was used in the alkylation, the reaction result was poor. The conversion of benzene and the selectivity of toluene were less than 61% (Entry 9), but the results were better than those of the zeolite catalyst.^29^ When IL [HO_3_S-(CH_2_)_3_-mim]Cl-FeCl_3 (*x*=0.67)_ was used in the alkylation, the benzene conversion increased to 71.6%, and the toluene selectivity reached 58.4% (Entry 12). It is seen that, with increase in Lewis acidic strength, the IL catalytic activity is enhanced. In conclusion, the synthesized double acidic ILs exhibit a high activity for some alkylation reactions, and the high catalytic performance may be attributed to the special double-acid characteristics. The research will be further in-depth studied.

**Table 5:** The benzene alkylation results over ILs^a^

Entry	Alkylation reagents	*N*_rea_	*T*/°C	*t*/h	Product	*C*/%	*S*/%
1	Propylene	4	240	0.25	Cymene	78.2	99.2
2	Propylene	10	240	0.25	Cymene	100	90.2
3	2-Butene	10	240	0.25	*sec*-Butylbenzene	100	85.0
4	1-Hexene	8	80	0.5	1-Methylpentylbenzene	100	72.5
5	1-Dodecene	8	40	0.5	2-Phenyl isomer	100	66.3
6	Benzyl chloride	10	60	0.5	Diphenylmethane	100	94.7
7	Bromoethane	4	240	2	Ethylbenzene	98.2	76.0
8	2-Chloropropane	4	80	2	Cymene	100	93.2
9	Methanol	4	240	4	Toluene	60.2	55.6
10^b^	Propylene	10	240	0.25	Cymene	76.0	95.4
11^c^	Propylene	10	240	0.25	Cymene	69.1	92.6
12^d^	Methanol	4	240	4	Toluene	71.6	58.4

a Benzene 5.0 g, IL [HO_3_S-(CH_2_)_3_-mim]Cl-ZnCl_2 (*x*=0.67)_ 0.25 g.

b The catalyst was IL [HO_3_S-(CH_2_)_3_-mim]Cl.

c The catalyst was [C_4_mim]Cl-ZnCl_2_.

d The catalyst was [HO_3_S-(CH_2_)_3_-mim]Cl-FeCl_3 (*x*=0.67)_. *N*_rea_: *n*(benzene) : *n*(alkylation reagent). *C*/%: the conversion of benzene. *S*/%: the selectivity of the product in the table.

## 4. Conclusions

The alkylation reaction of isobutane/2-butene was investigated in the presence of Brønsted–Lewis acidic ILs. IL [HO_3_S-(CH_2_)_3_-mim]Cl-ZnCl_2 (*x*=0.67)_ exhibited an outstanding catalytic performance with 100% conversion of 2-butene and 80.5% selectivity for TMP. The separated IL phase could be reused seven times without any obvious decrease in catalytic performance because of its stability and insolubility. The synergistic effect originating from the Brønsted and Lewis acid sites of IL and the water addition enhanced the IL catalytic performance significantly. Otherwise, the double acidic IL with good catalytic performance may have a potential applicability in other alkylation reactions.

## Conflicts of interest

There are no conflicts to declare.

## Supplementary Material

RA-008-C8RA03485K-s001
