# Supplementary material for: Isobutane/2-butene alkylation catalyzed by Brønsted–Lewis acidic ionic liquids
Source: RSC Adv. 2018 May 29;8(35):19551–9. doi: 10.1039/c8ra03485k (PMC9080696; doi:10.1039/c8ra03485k)
Supplement: RA-008-C8RA03485K-s001 [file RA-008-C8RA03485K-s001.pdf]

Electronic Supplementary Material (ESI) for RSC Advances.
This journal is © The Royal Society of Chemistry 2018

Supplementary materials

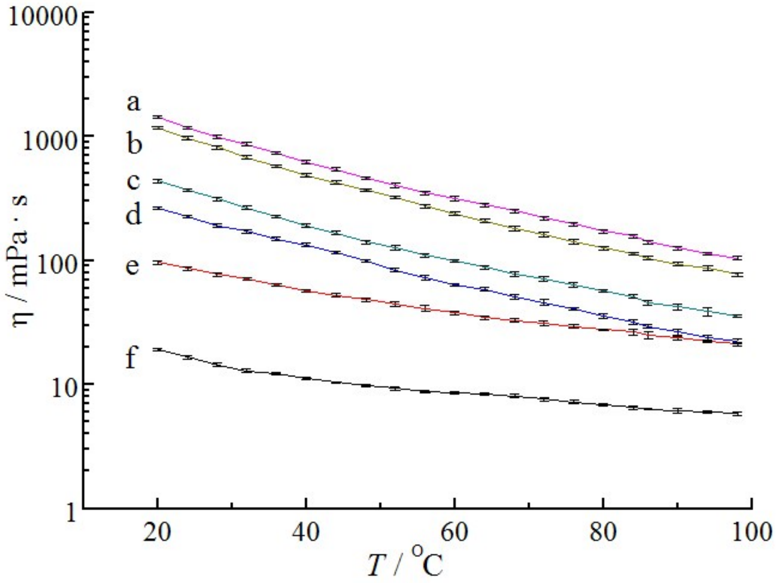

Fig. S1. Dynamic viscosity (η) of ILs. (a) [HO_3S–$(CH_2)_3$–mim]Cl–CuCl. (b) [HO_3S–$(CH_2)_3$–mim]Cl–$CuCl_2$. (c) [HO_3S–$(CH_2)_3$–mim]Cl–$FeCl_2$. (d) [HO_3S–$(CH_2)_3$–mim]Cl–$FeCl_3$. (e) [HO_3S–$(CH_2)_3$–mim]Cl–$ZnCl_2$. (f) [HO_3S–$(CH_2)_3$–mim]Cl–$ZnCl_2$ and water (the mass fraction of water was 30%). *x*(Lewis acidic metal chloride) = 0.67.

As showed in Fig. S1, the viscosity of IL decreased upon increasing the temperature. And the addition of the water obviously decreased the viscosity of the IL [HO_3S–$(CH_2)_3$–mim]Cl–$ZnCl_2$. Otherwise, the anions of the ILs also showed the large effect on the viscosities of the ILs, and these large differences in ILs viscosity as a function of the anion type show that the strength of anion−cation interactions have a pivotal role on ILs viscosity.

Table S1

The solubility of raw materials and products in the ILs [a]

Entry	ILs	2–Butane /g [b]	Isobutene /g [c]	Alkylate /g [d]
1	$[HO_3S–(CH_2)_3–mim]Cl$	1.28	6.40	2.16
2	$[C_4mim]Cl–ZnCl_2$	2.12	8.56	3.82
3	$[HO_3S–(CH_2)_3–mim]Cl–ZnCl_2$	1.86	6.23	3.20
4	$[HO_3S–(CH_2)_3–mim]Cl–CuCl_2$	1.70	5.85	2.89
5	$[HO_3S–(CH_2)_3–mim]Cl–CuCl$	1.52	5.21	2.65
6	$[HO_3S–(CH_2)_3–mim]Cl–AlCl_3$	1.33	5.60	2.41
7	$[HO_3S–(CH_2)_3–mim]Cl–FeCl_3$	1.84	6.32	3.46
8	$[HO_3S–(CH_2)_3–mim]Cl–FeCl_2$	1.66	6.69	2.80
9	$[HO_3S–(CH_2)_3–mim]Cl–ZnCl_2$ [e]	1.21	4.26	2.35
10	$[HO_3S–(CH_2)_3–mim]Cl–FeCl_3$ [e]	1.32	4.85	2.89
11	$[HO_3S–(CH_2)_3–mim]Cl–AlCl_3$ [e]	1.20	3.21	2.52

[a] The solubility mass of substances in 70 g ILs and 30 g water, x(Lewis acidic metal chloride) = 0.67. [b] T = -15 °C. [c] T = -5 °C. [d] T= 25 °C. [e] The solubility mass of substances in 100 g ILs.

As showed in Table S1, the solubility of 2-butene, isobutene or alkylate in the IL was poor. In comparison to the three substances, ILs had the strongest ability to dissolve 2-butene, attributing to the π–π complexes formation of IL and butane. Otherwise, the addition of water to IL improved the solubility of the substances in IL, which maybe improve the proton transfer and the formation of carbenium ions.

Table S2

Effect of the water addition on the acid strength of ILs [a]

Entry	ILs	Mass fraction of water /wt%				
		0	10	20	30	40
1	H_2SO_4	-11.95	/	/	/	/
2	$AlCl_3$	-2.60	/	/	/	/
3	$[HO_3S–(CH_2)_3–mim]Cl$	2.91	3.20	4.56	5.65	6.08
4	$[HO_3S–(CH_2)_3–mim]Cl–ZnCl_2$	1.26	1.86	2.40	3.02	4.21
5	$[HO_3S–(CH_2)_3–mim]Cl–CuCl_2$	2.52	3.04	3.86	5.52	6.43
6	$[HO_3S–(CH_2)_3–mim]Cl–CuCl$	2.86	3.65	4.50	6.84	7.02
7	$[HO_3S–(CH_2)_3–mim]Cl–AlCl_3$	-2.42	-2.70	0.21	0.98	1.65
8	$[HO_3S–(CH_2)_3–mim]Cl–FeCl_3$	0.82	1.38	2.16	2.96	3.70
9	$[HO_3S–(CH_2)_3–mim]Cl–FeCl_2$	1.65	2.22	3.40	4.60	5.36

[a] The indicator was *p*-nitroaniline, x(Lewis acidic metal chloride) = 0.67, T = 25 ^{o}C.

As showed in Table S2, the water addition decreased the IL acid strength by means of dilution and formation of a complex with metal ions. Otherwise, upon increasing of the acid strength of Lewis acidic metal chloride using to synthesize ILs, the acid strength of the obtained ILs enhanced.
